# Identification and Experimental Validation of Key Genes Related to Neuroendocrine in Periodontitis Based on Single‐Cell and Bulk Transcriptome Data

**DOI:** 10.1111/jcmm.71329

**Published:** 2026-08-23

**Authors:** Jiaguan Zhang, Ziyan Liu, Jianqing Deng, Peishan Huang, Huijuan Liu

**Affiliations:** ^1^ The Eighth Affiliated Hospital Sun Yat‐Sen University Shenzhe China; ^2^ Biological Laboratory of Hetao Cooperation Zone The Eighth Affiliated Hospital of Sun Yat‐Sen University Shenzhe China

**Keywords:** immune infiltration, key gene, neuroendocrine, periodontitis

## Abstract

Periodontitis is a chronic inflammatory condition linked to systemic diseases, yet the neuroendocrine mechanisms underlying its pathogenesis remain unclear. This study aimed to elucidate neuroendocrine‐related molecular pathways involved in periodontitis. Transcriptomic data and neuroendocrine‐related genes (NRGs) were integrated to identify candidate genes from the overlap between differentially expressed genes (DEGs) and NRGs. Machine learning, ROC analysis and expression validation were used to screen key genes and construct a diagnostic nomogram. Functional enrichment, immune infiltration, drug prediction, molecular docking and single‐cell analyses were performed. Key gene expression was further validated using clinical samples. Nine candidate genes were obtained from the intersection of 210 DEGs and 1441 NRGs. Five key genes—FOXN1, XBP1, HCLS1, IRF4 and RORA—were identified and used to develop an effective diagnostic model. These genes showed distinct tissue and cellular distributions and were enriched in pathways such as Ribosome and Protein processing in the ER. Sixteen immune cell types differed between groups, and 24 potential drugs, including melatonin, were predicted. Single‐cell analysis highlighted T cells and Endothelial cells as central cell populations. PCR results aligned with bioinformatic findings. Five neuroendocrine‐related genes were identified as potential diagnostic biomarkers and therapeutic targets, offering new insights into the neuroendocrine mechanisms of periodontitis.

**Abbreviations**
3D: three‐dimensional
AUC: area under the curve
BPs: biological processes
CCs: cellular components
cDNA: complementary DNA
DEGs: differentially expressed genes
DPSCs: dental pulp stem cells
FC: fold change
GEO: gene expression omnibus
GO: gene ontology
GSEA: gene set enrichment analysis
HC: healthy control
HVGs: highly variable genes
KEGG: kyoto encyclopedia of genes and genomes
LASSO: least absolute shrinkage and selection operator
MFs: molecular functions
MSigDB: molecular signatures database
NES: normalized enrichment score
NRGs: neuroendocrine‐related genes
PCA: principal component analysis
PCs: principal components
PD: periodontitis
PDLSCs: periodontal ligament stem cells
PPI: protein–protein interaction
QC: quality control
ROC: receiver operating characteristic curve
RT‐qPCR: reverse transcription‐quantitative polymerase chain reaction
scRNA‐seq: single‐cell RNA sequencing
SVM‐RFE: support vector machine—recursive feature elimination
VST: variance stabilizing transformation

## 1 Introduction

Periodontitis is a disease characterized by progressive destruction of the tooth‐supporting structures. Its local manifestations include gingival inflammation, bleeding on probing, loss of clinical attachment, periodontal pocket formation and ultimately tooth loss [1]. These features result from a dysbiotic microbial biofilm that initiates a host inflammatory immune response, modulated by environmental and genetic factors as well as microbial virulence. Beyond oral impacts, periodontitis is associated with increased risk of several systemic conditions, including adverse pregnancy outcomes, diabetes, cardiovascular and respiratory diseases, Alzheimer's disease and certain cancers [2]. As a highly prevalent condition, periodontitis poses a considerable public health burden [3]. Periodontal treatment employs interventions ranging from behavioural guidance to instrumentation, surgery and pharmacotherapy. While professional mechanical plaque removal shows significant effects [4], its effectiveness can vary due to deep probing depths and difficult‐to‐reach areas [4, 5]. Antimicrobials have been proposed to address these limitations, but concerns include bacterial resistance and adverse effects [6]. No single approach has proven superior [4], making it vital to investigate disease mechanisms and identify effective therapies.

Substantial evidence links periodontitis to neuroendocrine regulation disorders, particularly diabetes and depression [7, 8]. As the most prevalent neuroendocrine regulation disorder influencing periodontitis, diabetes promotes disease progression through multiple mechanisms, including oral microbiota dysbiosis, enhanced expression of inflammatory factors (e.g., IL‐1β, IL‐6, TNF‐α), disruption of the RANKL/OPG balance and AGE‐RAGE‐induced oxidative stress [9]. Similarly, depression may exacerbate periodontitis via stress‐mediated immunomodulation. For example, a clinical study by Haririan et al. demonstrated that individuals with major depressive disorder exhibited significantly elevated salivary levels of MMP‐8, IL‐1β and Chromogranin A, key biomarkers of periodontal tissue destruction, suggesting neuroendocrine‐immune crosstalk as a potential underlying mechanism [10]. More directly, the periodontal tissues are innervated by abundant sensory nerves and sympathetic nerves. Neuropeptides such as substance P released from nerve terminals can not only regulate the function of local immune cells, but also directly mediate osteoclast activation and bone resorption [11]. During this process, pro‐inflammatory neuropeptides secreted by sensory nerve terminals innervating periodontal tissues are recognized as one of the pivotal factors exacerbating local inflammation and alveolar bone destruction [12]. These findings demonstrate that the neuroendocrine system affects periodontal conditions not only indirectly via systemic diseases but also acts as a critical regulator of the local microenvironment, participating directly in the pathogenesis of periodontitis through the neuro‐immune‐bone metabolism crosstalk network. Nevertheless, there remains a lack of systematic understanding regarding the transcriptomic expression profiles, cellular localization and regulatory networks of neuroendocrine‐related genes in periodontitis lesions.

Single‐cell RNA sequencing (scRNA‐seq) offers unprecedented resolution compared to bulk RNA sequencing by enabling gene expression profiling at the single‐cell level. This technology significantly expands the capacity for cellular characterization, providing precise insights into transcriptional activity within individual cells and enabling researchers to uncover cellular heterogeneity, track dynamic changes in cell states and investigate individual variations in treatment response [13]. While scRNA‐seq application in periodontal research remains limited, key studies have begun to elucidate its potential. Lee et al. revealed that diabetes mellitus contributes to the deterioration of T cell and NK cell immunity, providing an immunological basis for the periodontitis‐diabetes association [14]. Chen et al. demonstrated the role of Ephrin‐Eph signalling in bone regeneration following initial periodontal therapy, highlighting its importance in periodontal healing [15]. Additionally, scRNA‐seq analysis of dental pulp stem cells (DPSCs) and periodontal ligament stem cells (PDLSCs) has revealed both shared and distinct transcriptional profiles, suggesting potential for both integrated regenerative strategies and tissue‐specific dental repair applications [16].

This study integrated periodontitis transcriptomic data from public databases and neuroendocrine‐related genes to identify key neuroendocrine‐associated genes using bioinformatics approaches. A diagnostic nomogram was developed and evaluated via calibration curves and decision curve analysis. Further analyses included gene correlation, chromosomal and subcellular localization, and gene–gene interaction networks. Enrichment analysis, immune infiltration profiling, drug prediction and molecular docking were conducted to elucidate molecular mechanisms. Cellular heterogeneity was explored at single‐cell resolution. Key gene expression was validated using RT‐qPCR on clinical samples. Our findings provide important insights into the neuroendocrine mechanisms in periodontitis and support the development of novel diagnostic and therapeutic strategies.

## 2 Methods

### 2.1 Acquisition of Data

The 3 periodontitis transcriptomic datasets utilized in this study were retrieved from the Gene Expression Omnibus (GEO, http://www.ncbi.nlm.nih.gov/geo). The GSE16134 dataset (platform: GPL570) included 241 gingival tissue specimens from periodontitis patients (diseased group) and 69 normal control tissue specimens (healthy group), which were used for differentially expressed gene screening and expression validation. The GSE223924 dataset (platform: GPL24676) contained 10 gingival tissue specimens from periodontitis patients (diseased group) and 10 normal control tissue specimens (healthy group), used for expression validation. The single‐cell GSE171213 dataset (platform: GPL24676) included periodontal tissue specimens from 5 patients with severe chronic periodontitis (PD group) and 4 healthy periodontal tissue specimens (HC group), used for single‐cell level analysis. Additionally, 1441 neuroendocrine‐related genes (NRGs) were retrieved from a published study [17] (Table S1).

### 2.2 Screening of Candidate Genes and Identification of Related Functions

Differentially expressed genes (DEGs) between the disease and health groups in the GSE16134 dataset were identified via the limma package (v 3.54.0) [18] with thresholds of |log_2_fold change (FC)| > 1 and *p*.adj < 0.05. A volcano plot visualizing DEGs was then generated via the ggplot2 package (v 3.4.1) [19] and expression heatmaps of the top 20 significantly upregulated and downregulated DEGs were constructed using the ComplexHeatmap package (v 2.14.0) [20]. Intersection genes between DEGs and neuroendocrine‐related genes (NRGs) were identified via the VennDiagram package (v 1.7.1) [21] and designated as candidate genes.

To explore the biological roles and signalling pathways of candidate genes in disease progression, functional enrichment analyses of biological processes and signalling pathways were performed relying on the Gene Ontology (GO) and Kyoto Encyclopedia of Genes and Genomes (KEGG) databases using the clusterProfiler package (v 4.2.2) [22] with *p*.adj < 0.05. GO terms were classified into 3 categories: biological processes (BPs), molecular functions (MFs) and cellular components (CCs), with the top 14 enriched pathways displayed according to the *p*.adj‐value ranking of enrichment. Additionally, a protein–protein interaction (PPI) network of candidate genes was plotted via the String online database (http://string‐db.org) (confidence > 0.15) to elucidate interrelationships between candidate genes at the protein level.

### 2.3 Screening of Key Genes and Construction of Diagnostic Nomogram

To screen for key neuroendocrine‐related genes in periodontitis, we first performed LASSO analysis on the candidate genes from the GSE16134 dataset using the glmnet package (v 4.1–8) [23]. The cv.glmnet() function was employed to conduct 10‐fold cross‐validation (parameters alpha = 1, family = binomial), using binomial deviance as the model performance metric. The λ value that minimized the cross‐validation deviance (lambda.min) was selected as the optimal parameter and feature genes were subsequently screened. In this process, to evaluate the internal stability of the LASSO model, we performed 1000 bootstrap resampling iterations on the GSE16134 training set. After each resampling, the area under the receiver operating characteristic curve (AUC) was calculated, and the distribution of the 1000 AUC values was used to assess model discrimination and stability. Subsequently, SVM‐RFE analysis was conducted on the candidate genes using the rfe() function from the caret package and the e1071 package (v 1.7–10) [24]. A radial basis kernel SVM (method = ‘svmRadial’) was adopted, and feature importance ranking was performed via caretFuncs. Starting from the full set of candidate genes, the least important gene was eliminated at each iteration, and the model was retrained on the remaining feature subset with accuracy evaluated through cross‐validation; this process was repeated until all genes were removed. The final number of retained features was determined by the subset achieving the highest cross‐validation accuracy. The VennDiagram package (v 1.7.1) was then used to identify the intersection of the feature genes selected by the two algorithms (LASSO and SVM‐RFE), and these were designated as candidate key genes.

Similarly, to further validate the robustness of the candidate key gene selection, the independent external dataset GSE10334 was employed for verification. Using the LASSO and SVM‐RFE model parameters and selection thresholds determined from the GSE16134 training set, feature selection was performed on the candidate genes in GSE10334 to examine the reproducibility of the selected genes.

Next, Wilcoxon tests (*p* < 0.05) were conducted in the GSE16134 and GSE223924 datasets to compare the expression levels and trends of candidate key genes. Receiver operating characteristic (ROC) curves for the candidate key genes were generated using the pROC package (v 1.18.0) [23] in both datasets. Finally, candidate key genes with significantly different expression levels, consistent expression trends and an area under the curve (AUC) value > 0.8 in ROC curves across both datasets were identified as key genes.

To assess the predictive utility of the identified key genes for periodontitis risk, a nomogram model was developed using the rms package (v 6.8–1) [24] in the GSE16134 dataset. By calculating the score for each key gene, the higher the total score, the greater the probability of disease occurrence. A calibration curve was plotted to validate the predictive accuracy and reliability of the model, where a slope closer to 1 indicates higher accuracy. Decision curve analysis (DCA) was performed using the rmda package (v 1.6) [25] to assess the clinical utility of the nomogram.

### 2.4 Key Gene Small Molecule Localization, Correlation Analysis and GeneMANIA Analysis

Understanding the chromosomal localization of key genes aids in elucidating their functions. The RCircos package (v 0.95) [26] was used to map the positions of key genes on human chromosomes. Subsequently, FASTA sequences of each key gene were collected from the NCBI database(https://www.ncbi.nlm.nih.gov/), and the CELLO online tool (http://cello.life.nctu.edu.tw/) was then employed to predict the distribution proportions of each key gene across subcellular structures, and tissue‐specific expression of each key gene was subjected to analysis via the BioGPS website (http://biogps.org/).

Subsequently, to investigate correlations among key genes, Spearman correlation analysis (|correlation coefficient (cor)| > 0.3, *p* < 0.05) was conducted via the psych package (v 2.1.6) [27] in the GSE16134 dataset. The corrplot package (v 0.92) [28] was employed to depict the results as a heatmap. Finally, the GeneMANIA database (https://genemania.org/) was used to construct a co‐expression network of key genes, allowing researchers to detect functionally interacting genes and explore their shared biological pathways.

### 2.5 Gene Set Enrichment Analysis (GSEA)

To elucidate the biological roles of each key gene in periodontitis progression, we performed GSEA. Initially, Spearman's rank correlation coefficients between each key gene and all other genes were computed using the psych package (v 2.1.6) in the GSE16134 dataset. The results were sorted in descending order to generate a pre‐ranked gene list for GSEA. Next, the c2.cp.kegg.v7.4.symbols.gmt gene set from the Molecular Signatures Database (MSigDB) (http://software.broadinstitute.org/gsea/msigdb/) was downloaded as the background gene set. GSEA was then conducted using the clusterProfiler package (v 4.2.2) with thresholds of |normalized enrichment score (NES)| > 1, false discovery rate (FDR) < 0.25 and *p* < 0.05.

### 2.6 Immune Infiltration Analysis

Periodontitis is intimately linked to immune cell infiltration and key gene expression. In the GSE16134 dataset, the CIBERSORT algorithm [29] was employed to quantify the infiltration levels of 22 immune cell types in disease and healthy group samples (excluding those with *p* > 0.05), aiming to characterize immune cell infiltration profiles. Subsequently, Wilcoxon signed‐rank tests were performed to evaluate differences in infiltration abundances of the 22 immune cell types between groups, identifying immune cells with significant differential infiltration (*p* < 0.05). Spearman correlation analysis was then conducted using the psych package (v 2.1.6) to assess associations between differentially infiltrated immune cells and between key genes and these cells (|cor| > 0.3, *p* < 0.05), with results visualized via the corrplot package (v 0.92).

### 2.7 Targeted Drugs and Molecular Docking of Key Genes

To further explore potential therapeutic drugs for periodontitis, we predicted drugs targeting key genes using the DGIdb database (https://www.dgidb.org/searchinteractions). A drug‐key gene interaction network was constructed using Cytoscape software (v 3.8.2). Meanwhile, investigating the interactions between key genes and related drugs can provide insights into disease therapeutic targets. Three‐dimensional (3D) protein structures of key genes were retrieved from the PDB database (http://www.rcsb.org) and converted to pdbqt format. The 3D structures of the two drugs with the highest intersection scores for key genes were separately retrieved from PubChem (https://pubchem.ncbi.nlm.nih.gov/). Molecular docking of proteins and active ingredients was then performed using AutoDock Vina (http://vina.scripps.edu/) to calculate binding free energy. The molecular docking results were visualized using the Discovery Studio package (v 4.1) [30]. Higher binding free energy indicates stronger affinity between the receptor and ligand, and a more stable complex conformation. A binding free energy more negative than −5.0 kcal/mol was considered a tight binding relationship.

### 2.8 Processing of Single‐Cell RNA Sequencing (scRNA‐Seq) Data and Screening of Key Cells

To obtain high‐quality data, quality control (QC) was performed on the single‐cell dataset GSE171213 using the Seurat package (v 4.3.0) [31]. The QC criteria were set as follows: percentage of mitochondrial RNA (percent.mt) < 25%; genes expressed in fewer than 3 cells, and cells with fewer than 200 expressed genes were excluded. Additionally, for HC1 and HC2, nFeature_RNA ≤ 1500 and nCount_RNA ≤ 6000; for HC3, nFeature_RNA ≤ 2000 and nCount_RNA ≤ 15,000; for HC4 and PD2, nFeature_RNA ≤ 2000 and nCount_RNA ≤ 10,000; for PD1 and PD3, nFeature_RNA ≤ 3000 and nCount_RNA ≤ 10,000; for PD4 and PD5, nFeature_RNA ≤ 3000 and nCount_RNA ≤ 15,000. QC results were visualized as violin plots. The data were subsequently normalized using the NormalizeData function. Subsequently, the variance stabilizing transformation (VST) method in the FindVariableFeatures function was used to identify the top 2000 genes with high intercellular variability, defined as highly variable genes (HVGs). Finally, the LabelPoints function was used for visualization, explicitly marking the top 10 HVGs.

Following this, all genes were subjected to scaling using the RunPCA function, and dimensionality reduction was performed on the top 2000 HVGs using principal component analysis (PCA) implemented in the same function. The statistical significance of principal components (PCs) was determined using the JackStrawPlot function (*p* < 0.05). The results of PCA dimensionality reduction were visualized using the JackStraw and Elbowplot functions to generate scree plots, which respectively showed the contributions of top‐ranked PCs to cell variability and assessed the statistical significance of each PC. Next, the FindNeighbors and FindClusters functions were applied to identify cell clusters (resolution = 0.5). The uUMAP method was used to cluster the principal components and partition cells into distinct cell clusters. Differentially expressed genes in each cell cluster were identified using the FindAllMarkers function (logFC = 0.5, min.pct = 0.2, only.pos = TRUE). Cell clusters were annotated by referencing marker genes reported in existing literature [15] and the CellMarker database (http://117.50.127.228/CellMarker/). To identify key cells in the single‐cell dataset GSE171213, a stacked bar plot was generated using the ggplot2 package (v 3.4.1) to display the proportion of each cell type in the disease and healthy groups. Subsequently, Wilcoxon rank‐sum tests were conducted to evaluate disparities in cell abundance between the disease and healthy groups (*p* < 0.05), followed by the identification of differentially abundant cells. Wilcoxon tests were then employed to compare the expression levels of key genes in these differentially abundant cells (*p* < 0.05). Cell types displaying significant expression differences for the majority of key genes were classified as key cells. Finally, the expression distribution of key genes within the key cells was visualized using UMAP plots.

### 2.9 Intercellular Communication Analysis and Pseudotime Trajectory Analysis

To investigate the interactions between key cells and other cell types during PD progression, we analysed intercellular communication among annotated cell types in the GSE171213 dataset. First, the CellChat package (v 1.6.1) [32] was used to explore intercellular signalling networks. Next, based on the CellPhoneDB database (https://www.cellphonedb.org/) and referencing CellChatDB.human, the celltalker method of the CellPhoneDB package (v 2.0) [33] was employed to identify significant receptor‐ligand interactions between annotated cell types (*p* < 0.05).

Subsequently, the key cells were clustered using the FindNeighbors and FindClusters functions in the Seurat package (v 4.3.0), and annotated into distinct cell subsets. Pseudotime trajectory analysis was then performed using the Monocle 2 package (v 2.26.0) [34] to visualize the differentiation trajectories of key cells. Furthermore, the plotPseudotimeHeatmap function was employed to illustrate the dynamic expression trends of key genes during cell differentiation.

### 2.10 Expression Analysis and the Reverse Transcription Quantitative PCR (RT‐qPCR)

For the study, gingival tissue samples were collected from periodontitis patients (*n* = 5) and healthy controls (*n* = 5) (samples collected from the Eighth Affiliated Hospital, Sun Yat‐sen University). Total RNA was extracted from each tissue using Trizol (Ambion) and then reverse‐transcribed into complementary DNA (cDNA) using the Hifair III 1st Strand cDNA Synthesis SuperMix. RT‐qPCR analysis was performed using 2× Universal Blue SYBR Green qPCR Master Mix. Primers for key genes were synthesized by Sangon Biotech (Shanghai, China) (Table S2). The GAPDH gene served as an endogenous reference gene. For every biological sample, 3 technical repetitions were carried out. Concurrently, the Ethics Committee granted approval for our study (IRB of the Eighth Affiliated Hospital, Sun Yat‐sen University). The resultant data underwent statistical analysis and were depicted via Graphpad Prism (v 8.0) [35].

### 2.11 Statistical Analysis

Statistical analyses were performed using R software (v 4.2.2). Intergroup differences were evaluated via the Wilcoxon test, with statistical significance defined as *p* < 0.05. Additionally, the Student's *t*‐test was employed to assess differences in RT‐qPCR experimental outcomes between groups.

## 3 Results

### 3.1 Functions of 9 Candidate Genes

**FIGURE 1:**
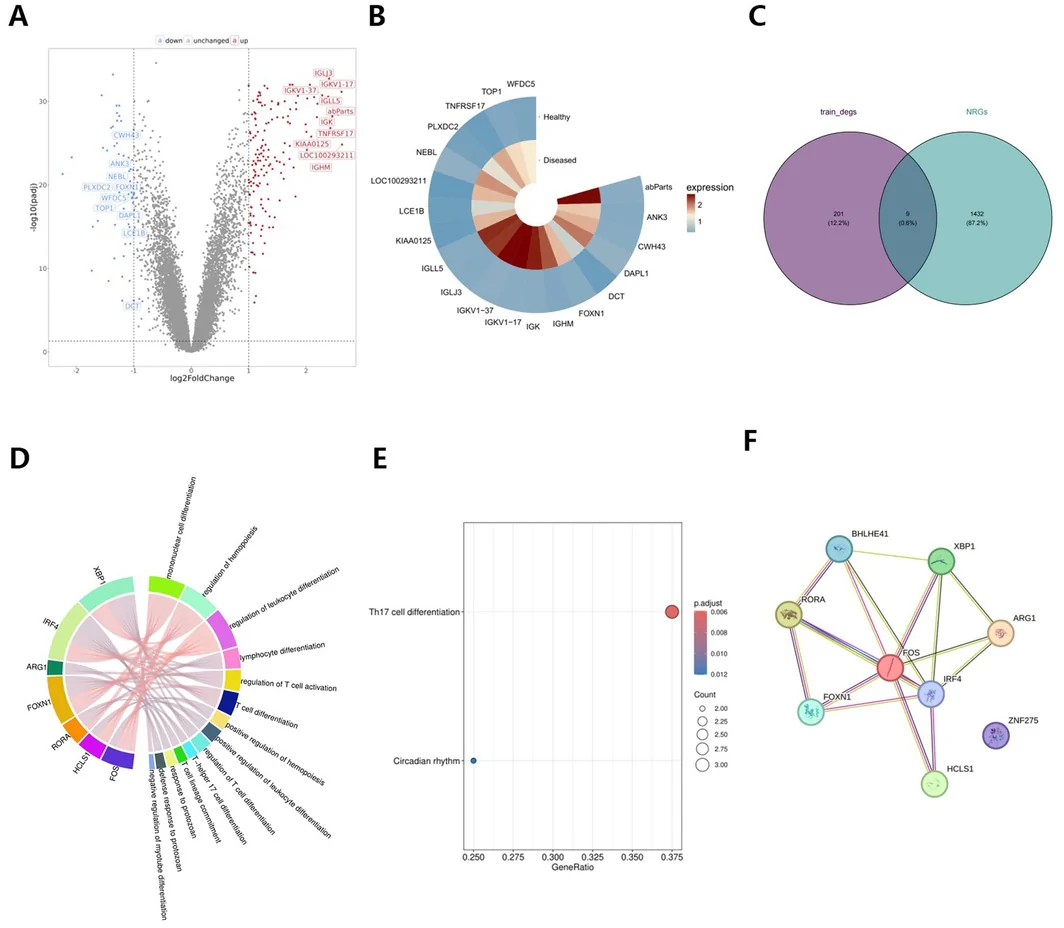
Functions of 9 candidate genes. (A) Volcano plot displaying 210 differentially expressed genes (DEGs) between disease and healthy groups in the GSE16134 dataset, including 54 upregulated genes (red) and 156 downregulated genes (blue). (B) Heatmap showing the expression patterns of the top 20 most significantly dysregulated DEGs, including IGLJ3, IGKV1‐17, IGKV1‐37, IGLL5 and others in disease and healthy groups. (C) Nine candidate genes that yield ed. by intersecting the 210 differentially expressed genes (DEGs) with 1441 neuroendocrine‐related genes (NRGs). (D) Bar plot showing GO biological process (BP) enrichment analysis results for the 9 candidate genes. The top 14 enriched pathways include regulation of leukocyte differentiation, regulation of haematopoiesis, T cell differentiation, etc. (*p*.adj < 0.05). (E) Two significantly enriched pathways identified by KEGG pathway analysis (*p*.adj < 0.05): Th17 cell differentiation and circadian rhythm. (F) PPI analysis revealed 16 interactions among 8 of the candidate genes, with the protein encoded by ZNF275 acting as an isolated node, while FOS and IRF4 exhibited frequent interactions with other genes.

A total of 210 differentially expressed genes (DEGs) were identified between the disease and healthy groups in the GSE16134 dataset via differential expression analysis, including 54 upregulated and 156 downregulated genes (Figure 1A). The top 20 most significantly dysregulated DEGs included IGLJ3, IGKV1‐17, IGKV1‐37, IGLL5, abParts, IGK, CWH43, TNFRSF17, KIAA0125, LOC100293211, ANK3, IGHM, NEBL, PLXDC2, FOXN1, WFDC5, TOP1, DAPL1, LCE1B and DCT (Figure 1B). Subsequently, intersecting the 210 DEGs with 1441 NRGs yielded 9 candidate genes (Figure 1C). These 9 candidate genes were significantly enriched in 91 GO terms (*p*.adj < 0.05), including 75 BPs and 16 MFs (Table S3). The top 14 enriched BPs included regulation of leukocyte differentiation, regulation of hemopoiesis, mononuclear cell differentiation, T cell differentiation, regulation of T cell activation, negative regulation of myotube differentiation, lymphocyte differentiation, regulation of T cell differentiation, defence response to protozoan, positive regulation of leukocyte differentiation, positive regulation of hemopoiesis, response to protozoan and T cell lineage commitment (Figure 1D). Additionally, KEGG pathway analysis identified 2 significantly enriched pathways (*p*.adj < 0.05) (Table S3) (Figure 1E): Th17 cell differentiation and circadian rhythm. PPI analysis revealed 16 interactions among 8 of the candidate genes, with the protein encoded by ZNF275 acting as an isolated node, while FOS and IRF4 exhibited frequent interactions with other genes (Figure 1F).

### 3.2 Identification of FOXN1, XBP1, HCLS1, IRF4 and RORA as Key Genes

**FIGURE 2:**
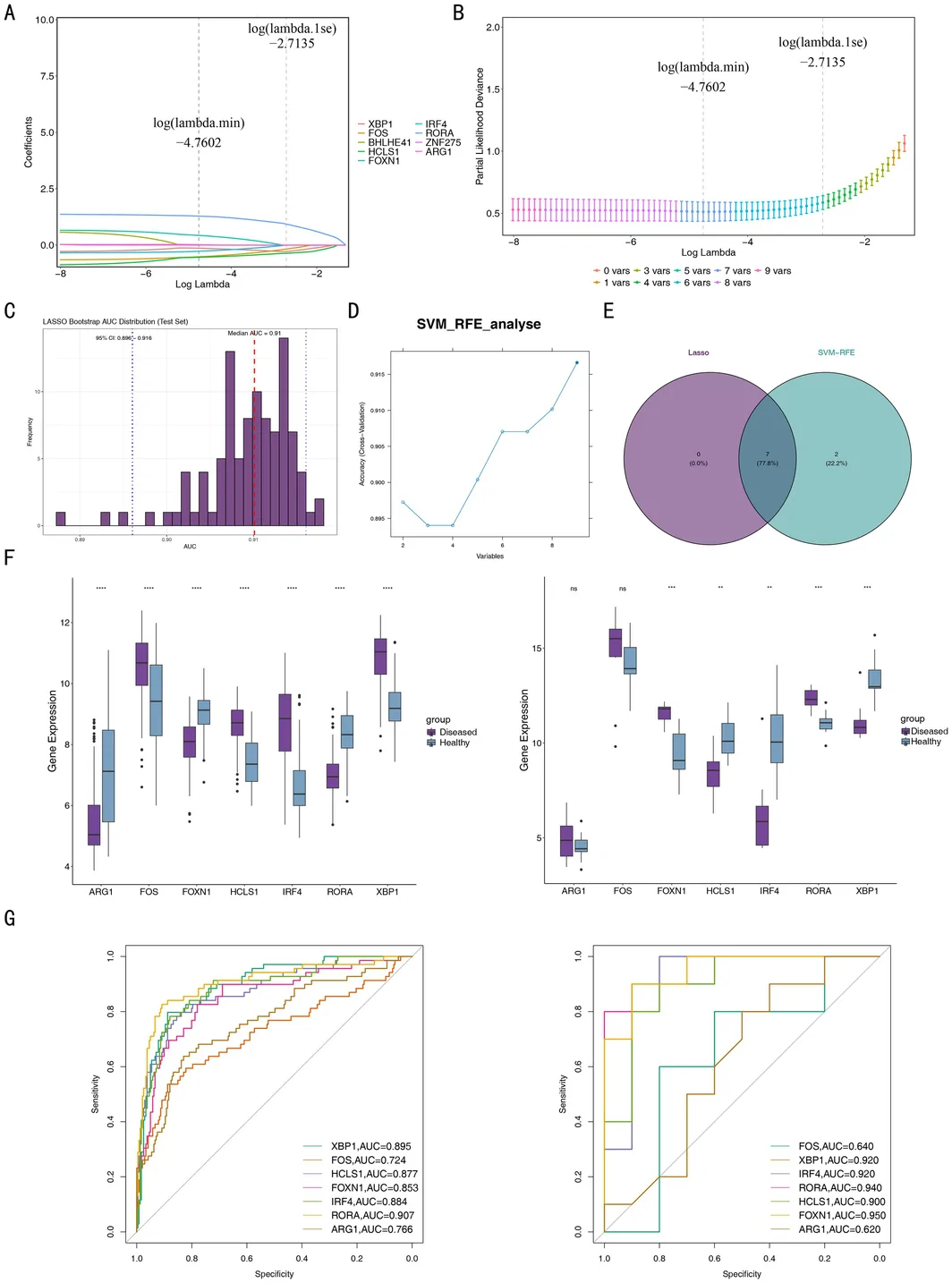
Identification of FOXN1, XBP1, HCLS1, IRF4 and RORA as key genes. (A, B) Seven characteristic genes were screened out through LASSO analysis: FOXN1, XBP1, HCLS1, IRF4, ARG1, FOS and RORA. (C) Bootstrap resampling results of LASSO analysis. (D) The SVM‐RFE model showed that the prediction model had the highest accuracy when all 9 genes (FOXN1, XBP1, HCLS1, IRF4, ARG1, FOS, RORA, BHLHE41 and ZNF 275) were retained. (E) Seven candidate key genes were identified by the overlap of characteristic genes through the two algorithms. (F) FOXN1, HCLS1, IRF4, XBP1 and RORA showed significant differential expression between the disease and healthy groups (*p* < 0.05), and FOXN1 and RORA were down‐regulated in the disease group; HCLS1, IRF4 and XBP1 were up‐regulated in the disease group. (G) The AUC values of the ROC curves for FOXN1, HCLS1, IRF4, XBP1 and RORA in both datasets were all > 0.8.

Through LASSO analysis, when log(lambda.min) was −4.7602 (lambda.min = 0.008563604), 7 characteristic genes were screened out: FOXN1, XBP1, HCLS1, IRF4, ARG1, FOS and RORA (Figure 2A,B). Meanwhile, the mean AUC of the LASSO model from 1000 bootstrap resampling iterations was 0.91, with a 95% confidence interval of [0.896, 0.916], indicating that the model had good discrimination ability and internal stability (Figure 2C). The SVM‐RFE model showed that the prediction model had the highest accuracy when all 9 genes were retained (Figure 2D). Therefore, the 9 candidate genes were included as characteristic genes in the subsequent analysis. Subsequently, the overlap of characteristic genes identified by the two algorithms revealed a total of 7 candidate key genes: FOXN1, XBP1, HCLS1, IRF4, ARG1, FOS and RORA (Figure 2E). In the independent external dataset GSE10334, feature selection was performed using the same LASSO and SVM‐RFE parameters and thresholds as those applied in the training set, and a total of six feature genes were obtained: XBP1, FOS, HCLS1, FOXN1, IRF4 and RORA, which were largely consistent with the candidate key genes identified in this study, further supporting the robustness of the machine learning‐based selection (Figure S1A–C).

In the two datasets, 5 genes, FOXN1, HCLS1, IRF4, XBP1 and RORA, showed extremely significant differential expression between the disease and healthy groups (*p* < 0.05), and the expression trends were consistent: FOXN1 and RORA were down‐regulated in the disease group, while HCLS1, IRF4 and XBP1 were up‐regulated in the disease group (Figure 2E). In addition, the AUC values of the ROC curves for these 5 genes in both datasets were all > 0.8 (Figure 2F). Therefore, FOXN1, HCLS1, IRF4, XBP1 and RORA were identified as key genes for periodontitis.

### 3.3 Construction and Evaluation of a Nomogram Based on 5 Key Genes

**FIGURE 3:**
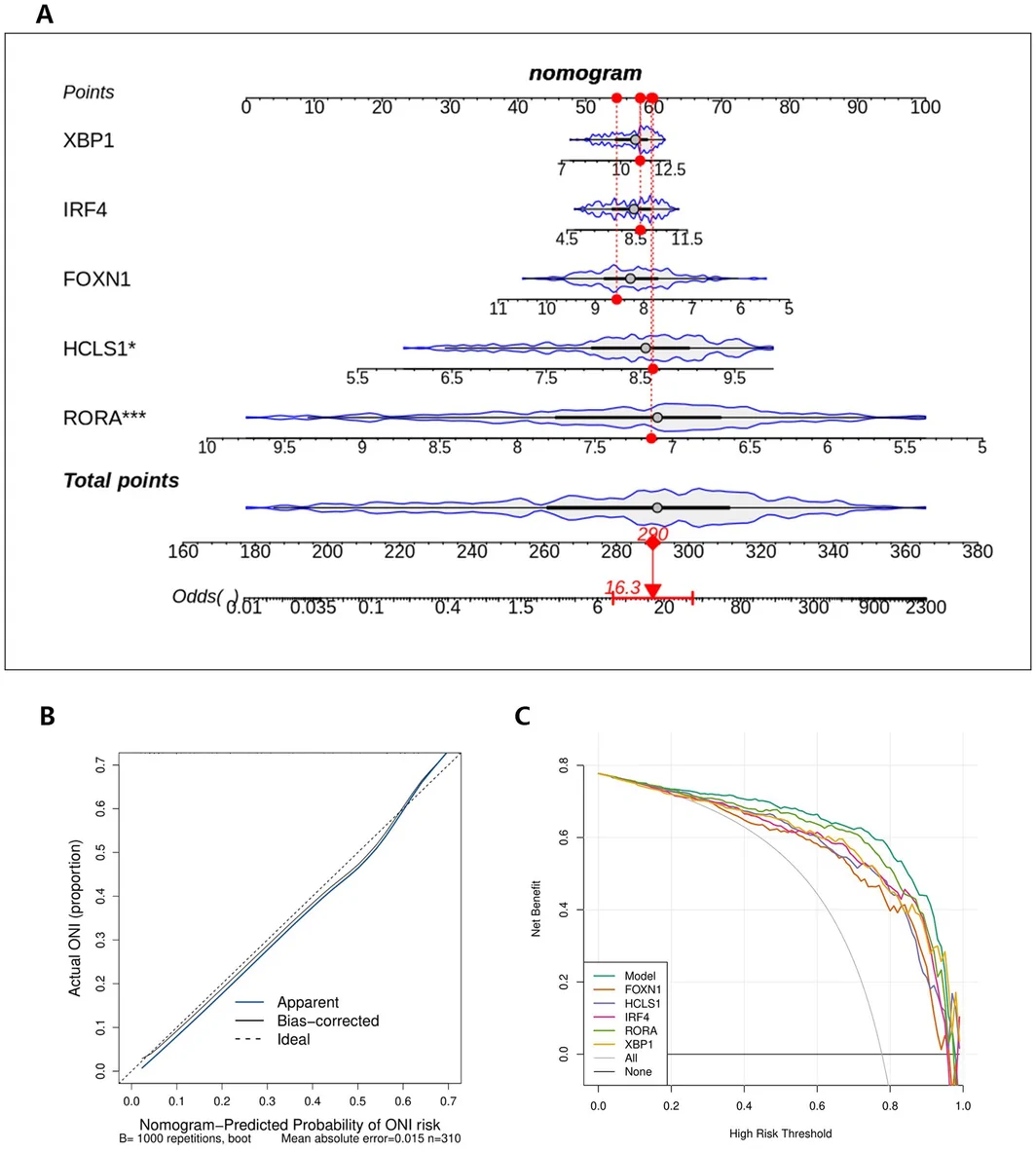
Construction and evaluation of a nomogram based on 5 key genes. (A) nomogram model was constructed. The total score of the 5 key genes (for FOXN1, HCLS1, IRF4, XBP1 and RORA) was 290, the prevalence of periodontitis was 16.3. (B) Calibration curve analysis demonstrated that the nomogram model constructed with key genes had minimal prediction errors, with a slope close to 1. (C) All key genes had scores above both the All line and None line.

A nomogram model was constructed using 5 key genes. When the total score of the 5 key genes was 290, the prevalence of periodontitis was 16.3 (Figure 3A). Calibration curve analysis demonstrated that the nomogram model constructed with key genes had minimal prediction errors, with a slope close to 1 (Figure 3B), indicating good predictive accuracy of the nomogram. Meanwhile, the DCA demonstrated that compared with individual key genes, the nomogram model provided greater clinical net benefit. All key genes had scores above both the All line and None line, suggesting the model has broad clinical application prospects (Figure 3C).

### 3.4 Small‐Molecule Localization and Functional Correlation of Key Genes

**FIGURE 4:**
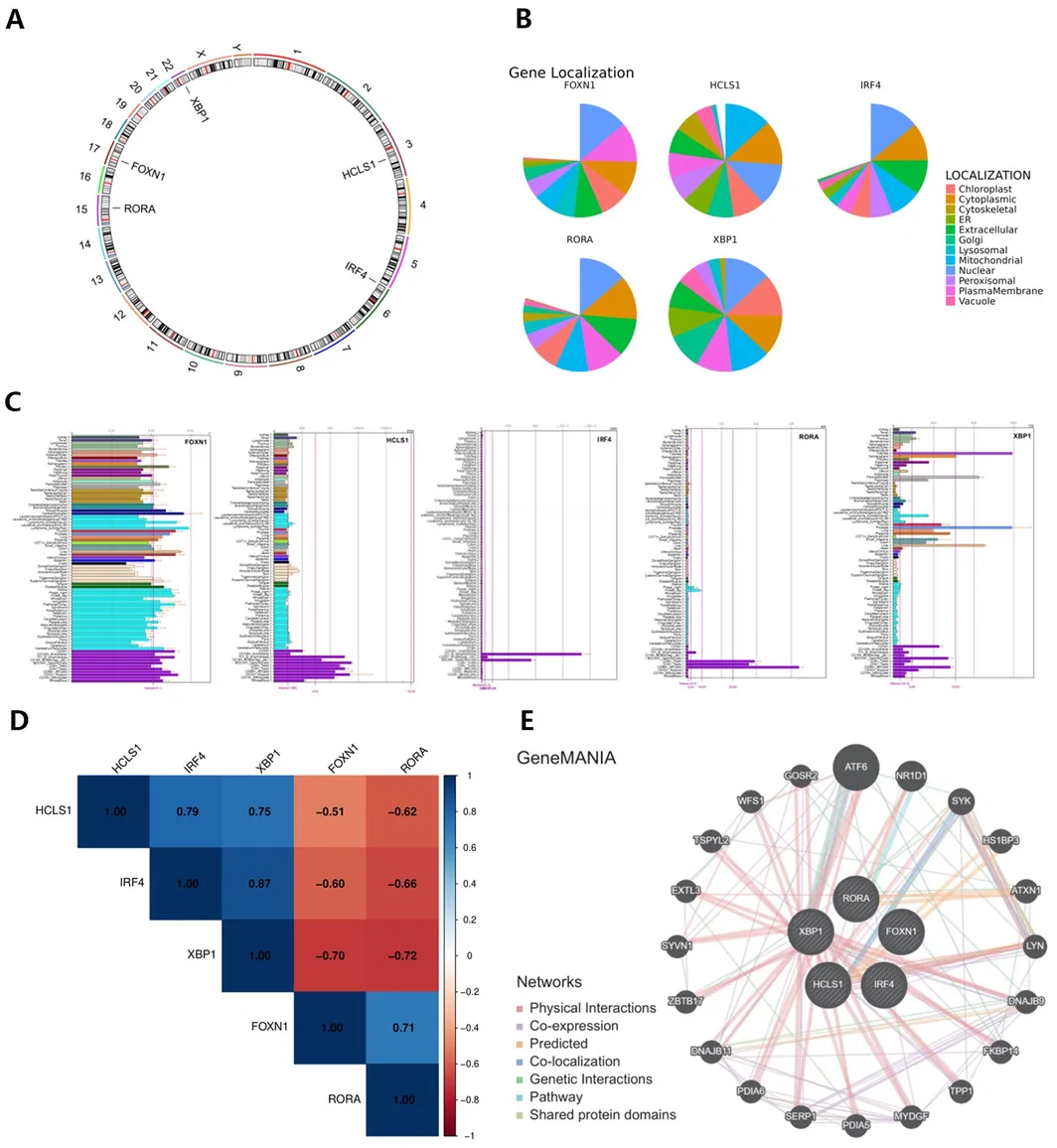
Small‐molecule localization and functional correlation of key genes. (A) Chromosomal localization analysis showed that HCLS1 was mapped to chromosome 3, IRF4 to chromosome 6, RORA to chromosome 15, FOXN1 to chromosome 17 and XBP1 to chromosome 22. (B) Subcellular localization revealed that FOXN1 was primarily distributed in the nucleus and plasma membrane, HCLS1 in the mitochondrial, nuclear and cytoplasmic compartments, IRF4 and RORA in the nucleus and cytoplasm, and XBP1 in the nucleus. (C1) Tissue‐specific expression profiling revealed that FOXN1 exhibited high expression across the majority of tissues, particularly in cardiac myocytes and Burkitt's lymphoma cells. (C2) HCLS1 was predominantly expressed in CD33+ myeloid cells and BDC44 dendritic cells. (C3) IRF4 was mainly expressed in BDCA4+ dendritic cells and 721 B lymphoblasts, (C4) RORA was enriched in CD56+ NK cells and CD8+ T cells. (C5) XBP1 exhibited specific expression in the prostate and trachea. (D) The 5 key genes demonstrated strong interconnections. Specifically, FOXN1 was positively correlated with RORA (cor = 0.71), HCLS1 with IRF4 (cor = 0.79) and IRF4 with XBP1 (cor = 0.87), whereas RORA showed a negative correlation with XBP1 (cor = −0.72). (E) GeneMANIA network analysis showing that the 5 key genes are associated with 20 homologous genes through physical interactions, co‐expression, in silico predicted interactions, subcellular co‐localization, genetic epistasis, shared biological pathways and common protein domains.

Chromosomal localization analysis showed that HCLS1 was mapped to chromosome 3, IRF4 to chromosome 6, RORA to chromosome 15, FOXN1 to chromosome 17 and XBP1 to chromosome 22 (Figure 4A). Subcellular localization revealed that FOXN1 was primarily distributed in the nucleus and plasma membrane, HCLS1 in the mitochondrial, nuclear and cytoplasmic compartments, IRF4 and RORA in the nucleus and cytoplasm, and XBP1 in the nucleus (Figure 4B). Tissue‐specific expression profiling revealed that FOXN1 exhibited high expression across the majority of tissues, particularly in cardiac myocytes and Burkitt's lymphoma cells. HCLS1 was predominantly expressed in CD33+ myeloid cells and BDC44 dendritic cells. IRF4 and RORA showed restricted tissue distribution: IRF4 was mainly expressed in BDCA4+ dendritic cells and 721 B lymphoblasts, while RORA was enriched in CD56+ NK cells and CD8+ T cells. XBP1 exhibited specific expression in the prostate and trachea (Figure 4C). The 5 key genes demonstrated strong interconnections. Specifically, FOXN1 was positively correlated with RORA (cor = 0.71), HCLS1 with IRF4 (cor = 0.79) and IRF4 with XBP1 (cor = 0.87), whereas RORA showed a negative correlation with XBP1 (cor = −0.72) (Figure 4D). Nevertheless, GeneMANIA network analysis demonstrated that the five key genes exhibited associations with 20 homologous gene counterparts through physical interactions, co‐expression, in silico predicted interactions, subcellular co‐localization, genetic epistasis, shared biological pathways and common protein domains, without identifying significant enrichment of biological processes (Figure 4E).

### 3.5 Enrichment Pathway Analysis of FOXN1, XBP1, HCLS1, IRF4 and RORA

**FIGURE 5:**
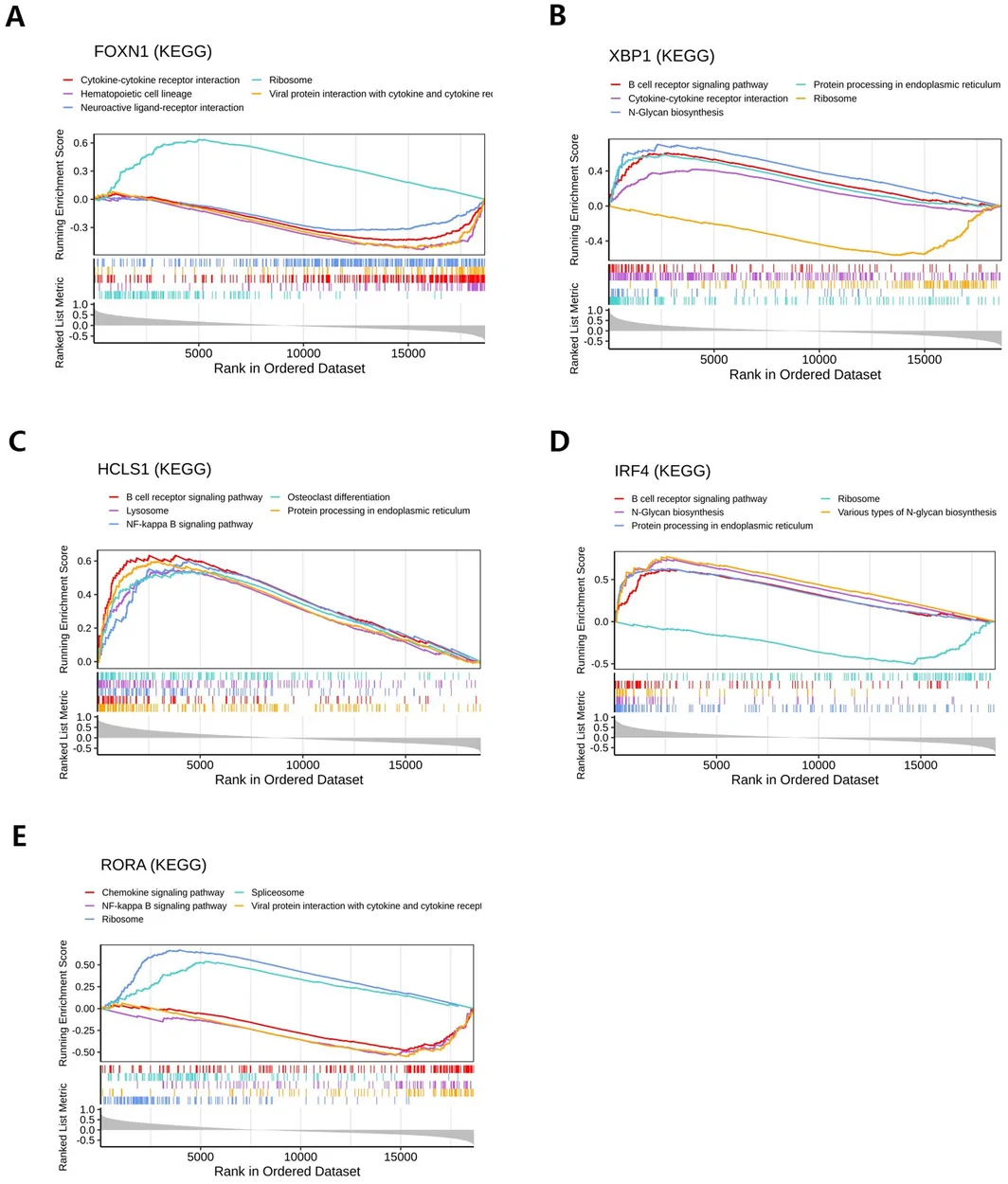
Enrichment Pathway Analysis of FOXN1, XBP1, HCLS1, IRF4 and RORA. (A) FOXN1 was enriched in 139 pathways, with the top 5 being Ribosome, Haematopoietic cell lineage, Cytokine‐cytokine receptor interaction, Viral protein interaction with cytokine and cytokine receptor and Neuroactive ligand‐receptor interaction. (B) XBP1 was enriched in 109 pathways, including Protein processing in endoplasmic reticulum, N‐Glycan biosynthesis, Ribosome, Cytokine‐cytokine receptor interaction and B cell receptor signalling pathway. (C) HCLS1 was enriched in 132 pathways, with the top 5 being Protein processing in endoplasmic reticulum, B cell receptor signalling pathway, NF‐κB signalling pathway, Lysosome and Osteoclast differentiation. (D) IRF4 was enriched in 96 pathways, including Protein processing in endoplasmic reticulum, N‐Glycan biosynthesis, Various types of N‐glycan biosynthesis, B cell receptor signalling pathway and Ribosome. (E) RORA was associated with 141 pathways, primarily Ribosome, Viral protein interaction with cytokine and cytokine receptor, NF‐κB signalling pathway, Spliceosome and Chemokine signalling pathway.

The biological functions of the 5 key genes were investigated based on the GSE16134 dataset (*p* < 0.05). Results showed that FOXN1 was enriched in 139 pathways, with the top 5 being Ribosome, Haematopoietic cell lineage, Cytokine‐cytokine receptor interaction, Viral protein interaction with cytokine and cytokine receptor, and Neuroactive ligand‐receptor interaction (Table S4, Figure 5A). XBP1 was enriched in 109 pathways, including Protein processing in endoplasmic reticulum, N‐Glycan biosynthesis, Cytokine‐cytokine receptor interaction and B cell receptor signalling pathway (Table S4, Figure 5B). HCLS1 was enriched in 132 pathways, with the top 5 being Protein processing in endoplasmic reticulum, B cell receptor signalling pathway, NF‐κB signalling pathway, Lysosome and Osteoclast differentiation (Table S4, Figure 5C). IRF4 was enriched in 96 pathways, including Protein processing in endoplasmic reticulum, N‐Glycan biosynthesis, Various types of N‐glycan biosynthesis and B cell receptor signalling pathway (Table S4, Figure 5D). RORA was associated with 141 pathways, primarily Ribosome, Viral protein interaction with cytokine and cytokine receptor, NF‐κB signalling pathway, Spliceosome and Chemokine signalling pathway (Table S4, Figure 5E). Among the top 5 enriched pathways, Ribosome, Protein processing in endoplasmic reticulum, B cell receptor signalling pathway, Cytokine‐cytokine receptor interaction and N‐Glycan biosynthesis were commonly enriched by multiple key genes.

### 3.6 Pathogenesis of PD Regulated by Multiple Immune Cells

**FIGURE 6:**
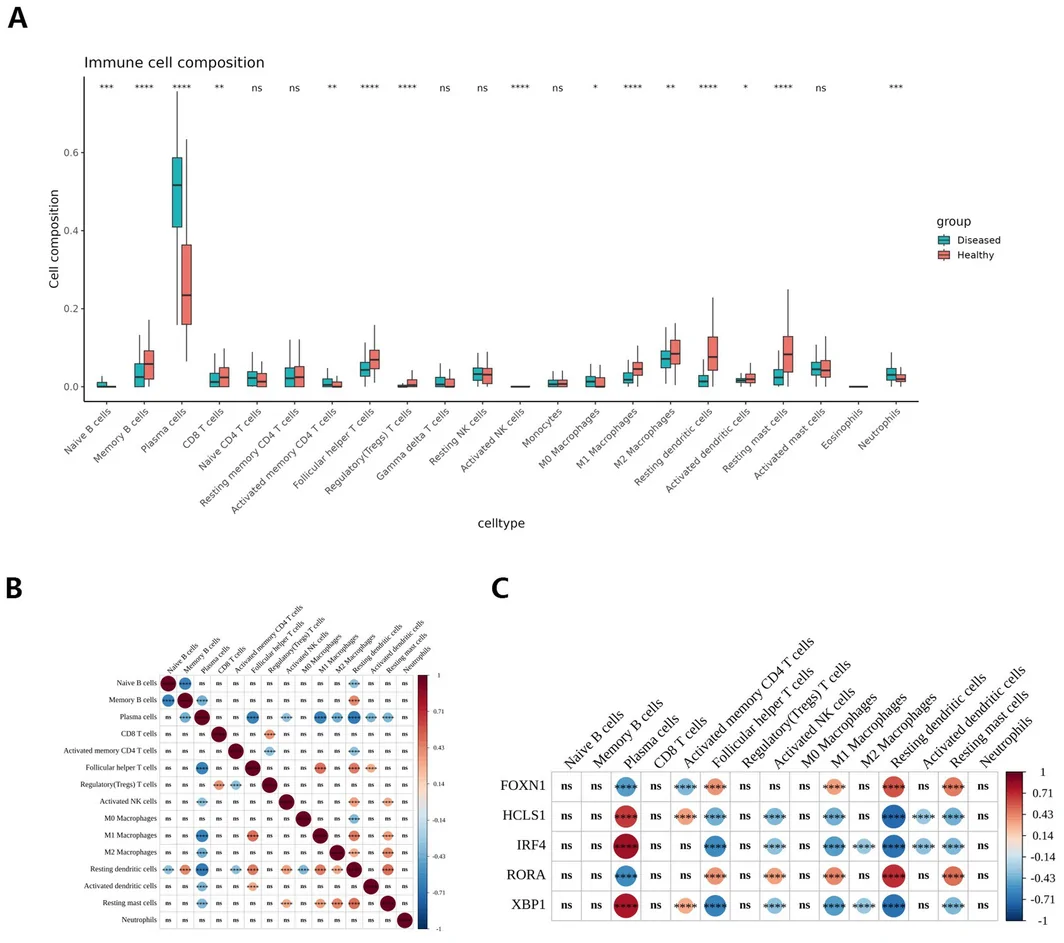
Pathogenesis of PD regulated by multiple immune cells. (A) Fifteen immune cell types exhibited significant infiltration differences (*p* < 0.05). Notably, plasma cells were markedly elevated in the disease group, whereas M2 macrophages, resting dendritic cells and resting mast cells were significantly more abundant in the healthy group. Eosinophils were absent in periodontitis samples. (B) Resting dendritic cells showed significant correlations with other cell types, particularly a strong negative correlation with plasma cells (cor < −0.3, *p* < 0.001). Plasma cells also exhibited significant associations, including negative correlations with follicular helper T cells and M1 macrophages (cor < −0.3, *p* < 0.001). Conversely, memory B cells showed a positive correlation with resting dendritic cells (cor > 0.3, *p* < 0.001) and M1 macrophages were positively correlated with follicular helper T cells (cor > 0.3, *p* < 0.001). (C) RF4, HCLS1 and XBP1 were positively correlated with plasma cells (cor > 0.3, *p* < 0.001), whereas FOXN1 and RORA showed negative correlations with plasma cells (cor > 0.3, *p* < 0.001). IRF4, HCLS1 and XBP1 were negatively correlated with resting dendritic cells (cor > 0.3, *p* < 0.001), while FOXN1 and RORA were positively correlated with resting dendritic cells.

Fifteen immune cell types exhibited significant infiltration differences (*p* < 0.05). Notably, plasma cells were markedly elevated in the disease group, whereas M2 macrophages, resting dendritic cells and resting mast cells were significantly more abundant in the healthy group. Eosinophils were absent in periodontitis samples (Figure 6A). Resting dendritic cells showed significant correlations with other cell types, particularly a strong negative correlation with plasma cells (cor < −0.3, *p* < 0.001). Plasma cells also exhibited significant associations, including negative correlations with follicular helper T cells and M1 macrophages (cor < −0.3, *p* < 0.001). Conversely, memory B cells showed a positive correlation with resting dendritic cells (cor > 0.3, *p* < 0.001), and M1 macrophages were positively correlated with follicular helper T cells (cor > 0.3, *p* < 0.001) (Figure 6B). Additionally, differentially infiltrated immune cells (plasma cells, follicular helper T cells, M1 macrophages, resting dendritic cells and resting mast cells) demonstrated significant correlations with the five key genes. IRF4, HCLS1 and XBP1 were positively correlated with plasma cells (cor > 0.3, *p* < 0.001), whereas FOXN1 and RORA showed negative correlations with plasma cells (cor > 0.3, *p* < 0.001). IRF4, HCLS1 and XBP1 were negatively correlated with resting dendritic cells (cor > 0.3, *p* < 0.001), while FOXN1 and RORA were positively correlated with resting dendritic cells (Figure 6C).

### 3.7 Prediction of Drugs Associated With Key Genes

**FIGURE 7:**
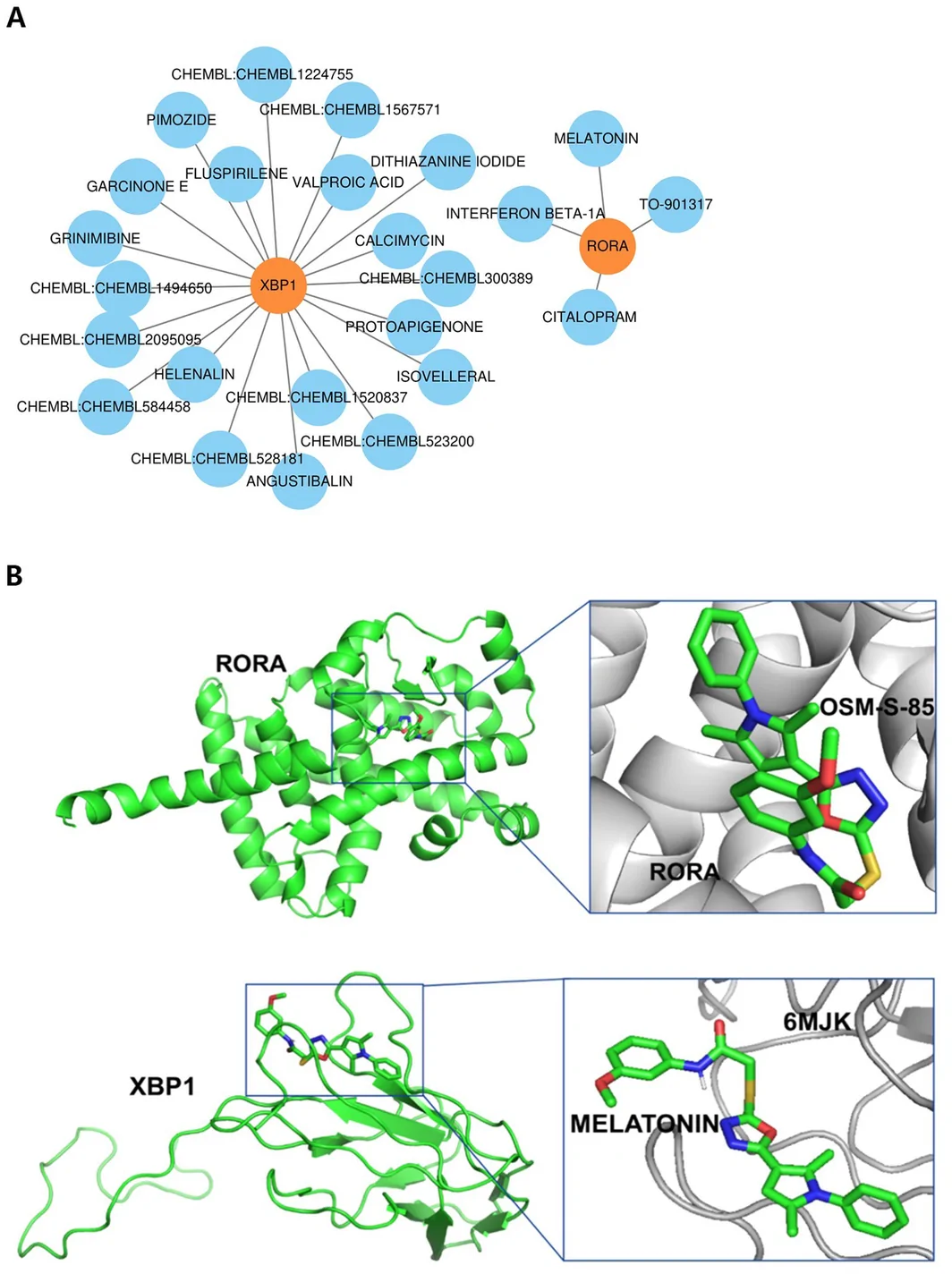
Prediction of drugs associated with key genes. (A) Twenty drugs were predicted for the XBP1 gene, while 4 drugs were predicted for the RORA gene. (B) RORA Molecular docking was performed using the drug with the highest intersection score for each gene: OSM‐S‐85 for RORA. XBP1 Molecular docking was performed using the drug with the highest intersection score for each gene: MELATONIN for XBP1.

Based on the DGIdb database, only 2 key genes were predicted to have associated drugs. Specifically, 20 drugs were predicted for the XBP1 gene, while 4 drugs were predicted for the RORA gene (Figure 7A). Molecular docking was performed using the drug with the highest intersection score for each gene: MELATONIN for XBP1 and OSM‐S‐85 for RORA. The binding energies were −6.86 kcal/mol for XBP1‐MELATONIN and −8.66 kcal/mol for RORA‐OSM‐S‐85, indicating favourable binding interactions that warrant further investigation as potential therapeutic agents (Figure 7B, Table 1).

**TABLE 1:** The binding energies of XBP1‐melatonin and RORA‐OSM‐S‐85.

Molecular	XBP1	RORA
MELATONIN	−6.86	—
OSM‐S‐85	—	−8.66

### 3.8 Identification of 11 Cell Types via Single‐Cell Analysis

**FIGURE 8:**
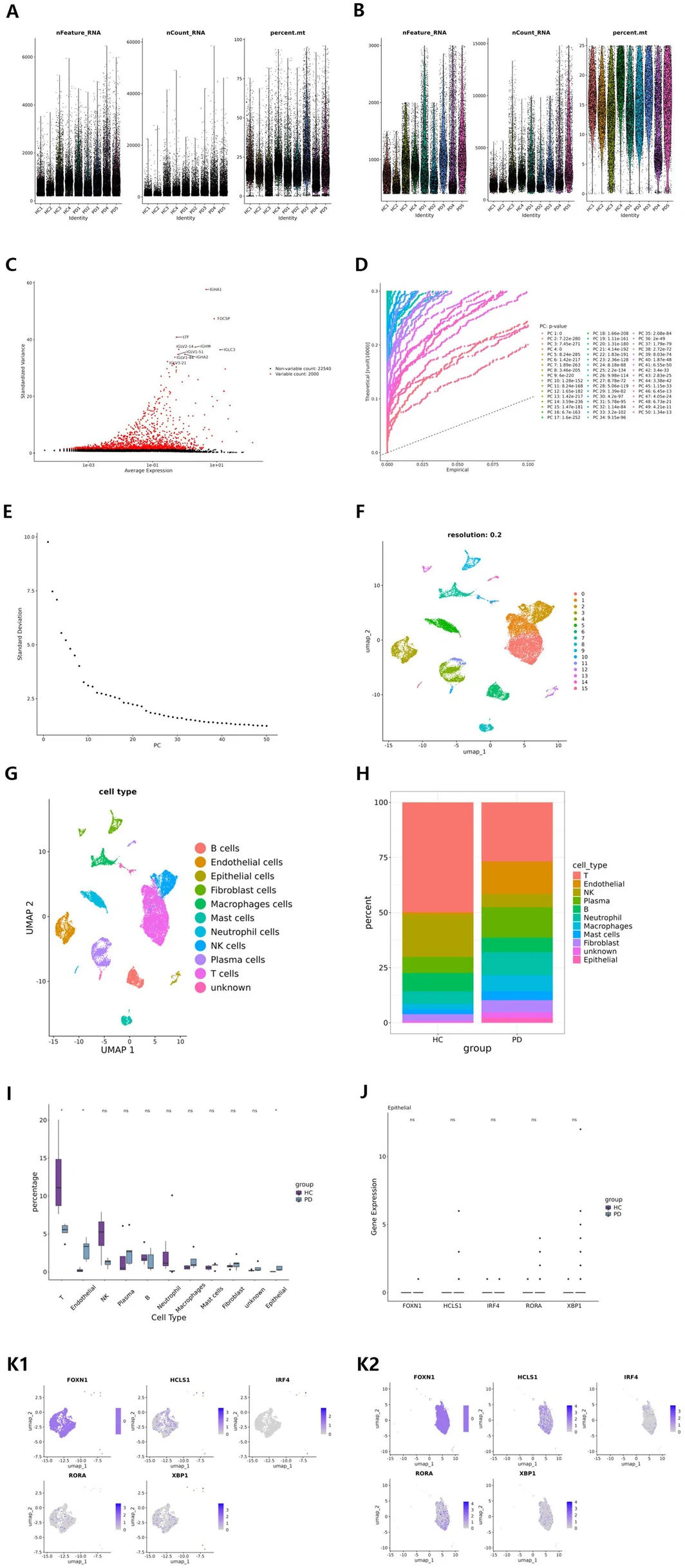
Identification of 11 cell types via single‐cell analysis. (A) First, QC was performed on the raw data of the single‐cell dataset GSE171213. Before QC, the dataset contained 37,665 cells and 24,540 genes. After QC filtering, 23,204 cells and 24,540 genes were retained. (B) First, QC was performed on the raw data of the single‐cell dataset GSE171213. Before QC, the dataset contained 37,665 cells and 24,540 genes. After QC filtering, 23,204 cells and 24,540 genes were retained. (C) The top 10 HVGs with the highest variability were IGHA1, FDCSP, LTF, IGLV2‐14, IGHM, IGLV1‐51, IGLC3, IGLV1‐44, IGHA2 and IGLV3‐21. (D) JackStraw plot showing the statistical significance of principal components (PCs). (E) Scree plot showing the contribution of the top 30 principal components to cell variability, with a plateau at the 30th PC. (F) UMAP dimensionality reduction clustering plot showing cells partitioned into 15 clusters. (G) Annotation by comparing differentially expressed genes with known marker genes identified 10 cell types and 1 unknown type: B cells, Endothelial cells, Epithelial cells, Fibroblasts, Macrophages, Mast cells, Neutrophils, NK cells, Plasma cells, T cells and unknown. (H) Among the 11 cell types, T cells, Endothelial cells and NK cells showed different proportions between disease and healthy groups. (I) T cells, Endothelial cells and Epithelial cells exhibiting significant differences (*p* < 0.05). (J) Five key genes showed significant expression differences in T cells and Endothelial cells (*p* < 0.05), designating these two cell types as key cells for periodontitis. (K1 and K2). endothelial cell FOXN1 was highly expressed in both key cell types. T cell FOXN1 was highly expressed in both key cell types.

First, QC was performed on the raw data of the single‐cell dataset GSE171213. Before QC, the dataset contained 37,665 cells and 24,540 genes. After QC filtering, 23,204 cells and 24,540 genes were retained (Figure 8A,B). The data were then normalized and scaled, and the top 2000 HVGs were selected based on the coefficient of variation. The top 10 HVGs with the highest variability were IGHA1, FDCSP, LTF, IGLV2‐14, IGHM, IGLV1‐51, IGLC3, IGLV1‐44, IGHA2 and IGLV3‐21 (Figure 8C).

PCA analysis of the 2000 HVGs showed a plateau at the 30th PC on the scree plot, thus selected for subsequent analysis (Figure 8A,D,E). Through dimensionality reduction and clustering, cells in GSE171213 were partitioned into 16 clusters (Figure 8F). Annotation by comparing differentially expressed genes with known marker genes identified 10 cell types and 1 unknown type: B cells, Endothelial cells, Epithelial cells, Fibroblasts, Macrophages, Mast cells, Neutrophils, NK cells, Plasma cells, T cells and unknown (Figure 8G). Among the 11 cell types, T cells, Endothelial cells and NK cells showed different proportions between disease and healthy groups (Figure 8H), with T cells, Endothelial cells and Epithelial cells exhibiting significant differences (*p* < 0.05) (Figure 8I). Additionally, 5 key genes showed significant expression differences in T cells and Endothelial cells (*p* < 0.05), designating these two cell types as key cells for periodontitis (Figure 8J). FOXN1 was highly expressed in both key cell types (Figure 8K).

### 3.9 Cell Communication of Annotated Cells and Pseudotime Analysis of Key Cells

**FIGURE 9:**
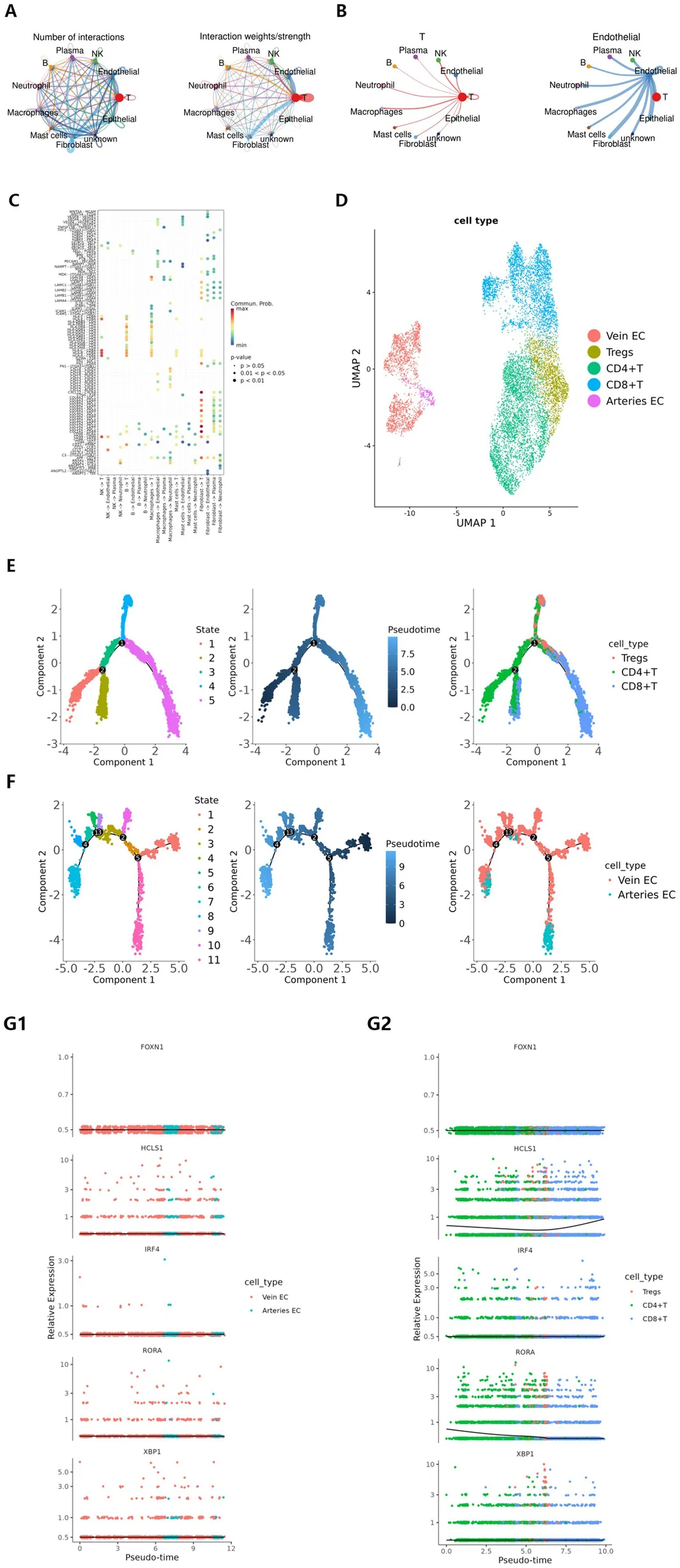
Cell communication of annotated cells and pseudotime analysis of key cells. (A) In the intercellular communication network of all annotated cells, within the disease group, both the number and intensity of information exchange between T cells and Endothelial cells with other cells were higher than those of other cell types. (B) The communication intensity between T cells and Endothelial cells was stronger than that between other cell pairs. (C) Bubble plot showing major ligand‐receptor interactions in the disease group. The information exchange intensities of B cells→T cells, Macrophages→T cells, NK cells→T cells and Fibroblasts→T cells, along with their ligand‐receptor interactions, are relatively high. (D) Subsequent secondary clustering of T cells and Endothelial cells (key cell types) annotated T cells into three subtypes: Tregs, CD4+ T cells and CD8+ T cells; Endothelial cells were annotated into two subtypes: Vein EC and Arteries EC. (E) Pseudotime trajectory analysis of T cells showing the differentiation process divided into 5 states. CD4+ T cells are mainly present in the initial stage, while CD8+ T cells are in the terminal stage. (F) Pseudotime trajectory analysis of Endothelial cells showing the differentiation process divided into 11 states. Vein EC is mainly present in the initial stage, and Arteries EC in the terminal stage. (G1) Heatmaps showing the dynamic expression trends of 5 key genes during T cell and Endothelial cell differentiation. HCLS1 gene shows a decline followed by high expression during T cell development, while RORA continuously decreases during differentiation. The other 3 key genes are lowly expressed. All 5 key genes are lowly expressed during Endothelial cell differentiation. (G2) The T cell HCLS1 gene showed a decline followed by high expression during T cell development, while RORA continuously decreased during differentiation. The other 3 key genes were lowly expressed. All 5 key genes were lowly expressed during Endothelial cell differentiation.

In the intercellular communication network of all annotated cells, within the disease group, both the number and intensity of information exchange between T cells and Endothelial cells with other cells were higher than those of other cell types (Figure 9A). Moreover, the communication intensity between T cells and Endothelial cells was stronger than that between other cell pairs (Figure 9B). Additionally, within the disease group, the information exchange intensities of B cells→T cells, Macrophages→T cells, NK cells→T cells and Fibroblast→T cells, along with their ligand‐receptor interactions, were relatively high (Figure 9C).

Subsequent secondary clustering of T cells and Endothelial cells (key cell types) annotated T cells into three subtypes: Tregs, CD4+ T cells and CD8+ T cells; Endothelial cells were annotated into two subtypes: Vein EC and Arteries EC (Figure 9D). Pseudotime trajectory analysis showed that T cells differentiated from an initial developmental state, forming a root structure with three branching trajectories. The differentiation process was divided into five states: State 1 dominated the initial stage, State 3 represented the intermediate stage, and States 2, 4 and 5 corresponded to the terminal differentiation stage. CD4+ T cells were mainly present in the initial stage, while CD8+ T cells were in the terminal stage (Figure 9E). Endothelial cells differentiated from the initial state, primarily forming a root structure with two branching trajectories. The process was divided into 11 states: State 1 dominated the initial stage, and States 11 and 7 corresponded to the terminal stage. Vein EC was mainly present in the initial stage, and Arteries EC in the terminal stage (Figure 9F). Notably, the HCLS1 gene showed a decline followed by high expression during T cell development, while RORA continuously decreased during differentiation. The other 3 key genes were lowly expressed. All 5 key genes were lowly expressed during Endothelial cell differentiation (Figure 9G).

### 3.10 Expression Analysis of Key Genes

**FIGURE 10:**
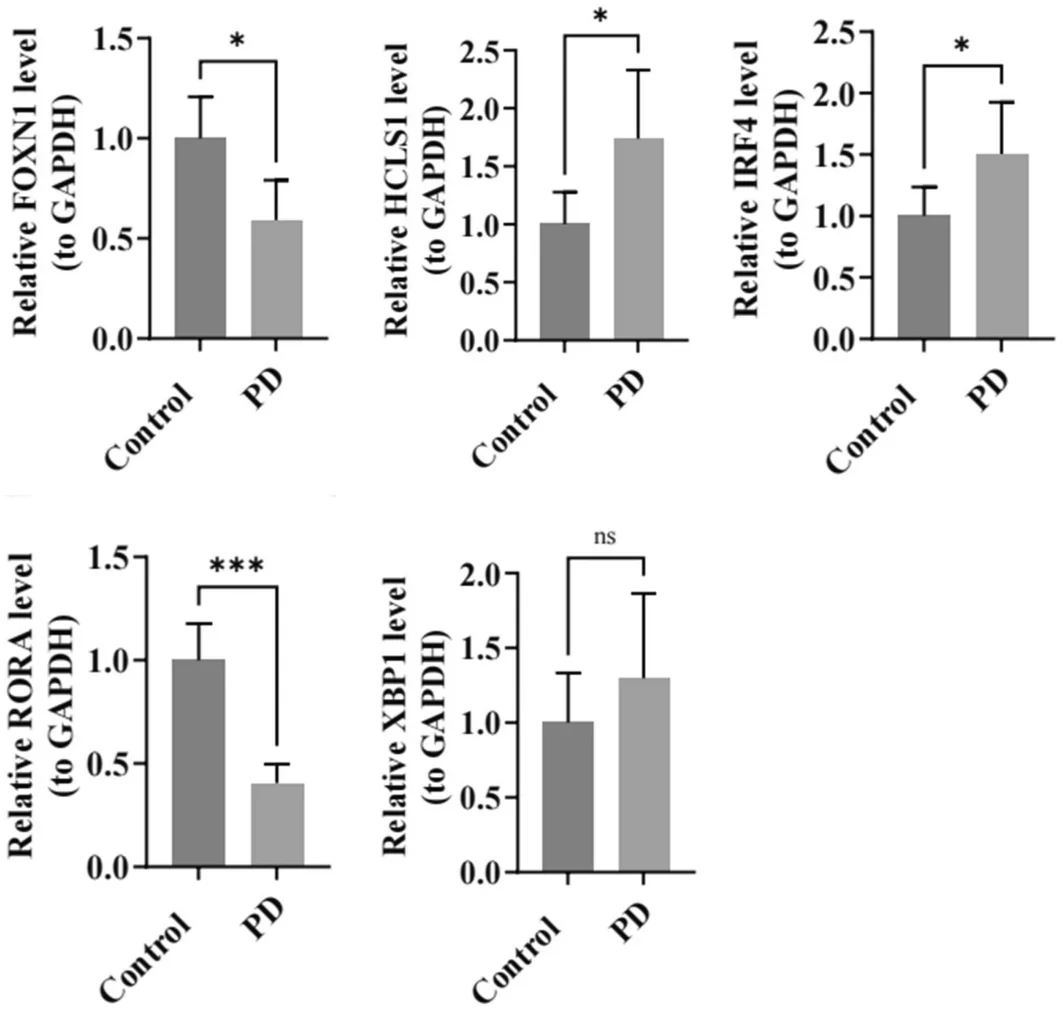
Expression analysis of key genes. Bar graphs showing RT‐qPCR validation results of 5 key genes in clinical samples. Compared with the control group, FOXN1 and RORA are downregulated in the disease group (*p* < 0.05) and HCLS1 and IRF4 are upregulated in the disease group (*p* < 0.05), consistent with bioinformatics analysis results. XBP1 also shows an upregulation trend in the disease group, but the difference is not statistically significant. Sample size: Disease group *n* = 5, control group *n* = 5. Data are expressed as mean ± SD. *p < 0.05, **p < 0.01, ***p < 0.001, ns, not significant.

PCR experiments showed that FOXN1 and RORA were downregulated in the disease group compared to the control group in clinical samples, with significantly different expression levels (*p* < 0.05), consistent with the results of bioinformatics analysis. HCLS1 and IRF4 were upregulated in the disease group, and the expression levels were significantly different from those in the control group (*p* < 0.05), which was also consistent with the bioinformatics results. XBP1 was also upregulated in the disease group, but the difference was not significant. The overall expression trend was consistent with our research, which might be related to the sample collection period or experimental operation (Figure 10).

## 4 Discussion

Periodontitis is a chronic inflammatory disease characterized by the progressive destruction of periodontal tissues [1]. It is initiated by dysbiotic microbial biofilms and driven by a dysregulated host immune response, influenced by genetic and environmental factors. The disease is also linked to systemic conditions like diabetes, cardiovascular diseases and Alzheimer's disease, contributing to a significant public health burden [2]. Current treatments, primarily mechanical debridement and antimicrobials, face limitations such as limited anatomical access, adverse effects and antimicrobial resistance [4]. Although associations between periodontitis and neuroendocrine disorders like diabetes and depression are recognized [7], the underlying genetic mechanisms remain poorly understood, highlighting the need for novel therapeutic targets. In this investigation, we identified five pivotal genes—FOXN1, HCLS1, IRF4, XBP1 and RORA—associated with periodontitis. Functional analyses revealed that these genes are involved in five major pathways: ribosome, protein processing in endoplasmic reticulum, B cell receptor signalling pathway, cytokine‐cytokine receptor interaction and N‐glycan biosynthesis.

### 4.1 The Distinct Roles of Key Genes in Periodontitis

The FOXN1 gene, encoding a transcription factor with a Forkhead Box DNA‐binding domain, is crucial for immune tolerance and preventing autoimmunity [36]. While no prior studies directly link FOXN1 to periodontitis, our work is the first to establish this association. We hypothesize that its role involves T‐cell deficiency and neuroendocrine stress pathways. Reduced FOXN1 expression impairs thymic development, leading to decreased T‐cell numbers [37]. Since regulatory T cells (Tregs) are vital for controlling periodontal pathogens, a T‐cell deficit may compromise pathogen clearance and facilitate disease progression [38]. Furthermore, the established link between depression and periodontitis may be mediated by FOXN1; chronic stress elevates glucocorticoids [39], which downregulate FOXN1 [40]. Consistent with this, we observed significantly lower FOXN1 expression in periodontitis tissues, supporting its potential as a biomarker.

Another key gene identified in our study is XBP1, a central transcription factor in the unfolded protein response (UPR) that regulates endoplasmic reticulum (ER) homeostasis and is implicated in inflammation [41]. Accumulating evidence confirms the involvement of XBP1 in periodontitis pathogenesis. Knocking down XBP1 in rat models alleviated tissue damage, inflammation and bone loss by restoring the Th17/Treg balance via suppression of IL‐17 signalling [42]. Additionally, XBP1 is upregulated in patients with concurrent diabetes and periodontitis, where hyperglycemia‐induced ER stress impairs the osteogenic potential of periodontal ligament stem cells [43]. Our data corroborate significant XBP1 upregulation in periodontitis, underscoring its pathogenic role and therapeutic potential.

A third key gene identified in our analysis is HCLS1, which is essential for haematopoietic cell function with critical roles in neutrophils [44]. It was previously identified as a master regulator in periodontitis through computational analysis [45], a finding that we confirm by demonstrating its upregulation in our dataset. While direct causal evidence is lacking, we propose several mechanistic hypotheses: (1) HCLS1 overexpression enhances neutrophil chemotaxis and activation, causing sustained inflammation and tissue damage [46]; (2) It activates the TLR4/MyD88/NF‐κB axis, boosting pro‐inflammatory cytokine production (e.g., TNF‐α, IL‐1β, IL‐6; [47]); and (3) It stimulates the PI3K/Akt pathway, promoting a pro‐inflammatory M1 macrophage shift that exacerbates bone loss [48]. Experimental validation of these mechanisms is a future goal.

The fourth gene, IRF4, is a transcriptional regulator significantly upregulated in periodontitis [45]. Mechanistically, it negatively regulates the osteogenic differentiation of periodontal ligament stem cells via the IL‐18 pathway [49]. Moreover, IRF4 is critical for RANKL‐induced osteoclastogenesis and Th17 cell differentiation [38], both key processes in inflammatory bone resorption. Thus, IRF4 appears to promote periodontitis by enhancing osteoclast activity and amplifying destructive immune responses.

Finally, RORA is a nuclear receptor expressed in various tissues, including immune cells [50]. Its dysregulation in periodontitis [45] contributes to pathogenesis through multiple mechanisms: (1) facilitating NF‐κB nuclear translocation to drive pro‐inflammatory cytokine and chemokine production [51]; (2) under normal conditions, RORA inhibits osteoclast differentiation via RANKL signalling; however, its downregulation in periodontitis promotes bone resorption; and (3) acting as a negative regulator of B cell proliferation [52], and consequently, its downregulation leads to B cell expansion and enhanced osteoclast formation.

### 4.2 Synergistic Integrated Regulation of Key Genes

Based on available studies, we hypothesize that the functions of the aforementioned genes do not act independently. Instead, they collectively form a dysregulated network initiated by neuroendocrine stress, which ultimately drives immune activation and bone destruction. Under the condition of systemic neuroendocrine disorders such as chronic psychological stress, persistently elevated glucocorticoids can downregulate the expression of FOXN1 [44]. Similarly, significant downregulation of RORA has also been observed in animal models of post‐traumatic stress disorder, a psychological stress‐associated disease [53].

FOXN1 supports T cell development by regulating the formation and proliferation of thymic epithelial cells, and its decreased expression can lead to immunodeficiency [54]. RORA has also been verified to exert a vital regulatory effect on T cells [55]. Accordingly, we propose that the downregulation of FOXN1 and RORA induced by neuroendocrine disorders may jointly impair the body's protective mechanisms. Impaired T cell development may reduce immune surveillance capacity, thereby attenuating the inhibitory effect on osteoclast differentiation [56].

Meanwhile, excessive activation of XBP1 and IRF4 can promote Th17 cell differentiation [48, 57], which sustains chronic inflammation and mediates tissue destruction during periodontitis [58]. In addition, as a key scaffold protein of the TLR4/NF‐κB signalling pathway, HCLS1 may aggravate the release of pro‐inflammatory cytokines [55] and further amplify inflammatory signals.

It is thus speculated that these five genes may collaboratively establish a pro‐inflammatory microenvironment in response to neuroendocrine stimulation, synergistically triggering the imbalance of T cell subsets and excessive osteoclast activation, and ultimately facilitating the progression of periodontitis. This hypothetical model links neuroendocrine signals with immune activation and tissue destruction, which is consistent with the pivotal role of T cells identified in our subsequent single‐cell analysis. It provides a verifiable preliminary framework for further exploring the regulatory mechanisms of the neuroendocrine‐immune‐bone metabolism axis in periodontitis.

### 4.3 The Five Hub Genes Commonly Enriched Several Pathways Directly or Indirectly Linked to Periodontitis

Functional enrichment analysis revealed that the five key genes collectively participate in multiple interconnected pathways critical to periodontitis pathogenesis, including ribosomal biogenesis, ER stress response, immune signalling and post‐translational modification processes. The Ribosome Pathway and ER Protein Processing Pathway represent fundamental cellular housekeeping functions that are disrupted in periodontitis. The ribosome pathway is enriched for FOXN1 and RORA, with RORA directly promoting ribosomal gene transcription [57]. However, lipopolysaccharide from periodontal pathogens suppresses ribosomal function in osteoblasts, disrupting bone homeostasis and contributing to alveolar bone loss [59]. The ER protein processing pathway is enriched for XBP1 and IRF4. XBP1 serves as a central regulator maintaining ER proteostasis [60], while IRF4 indirectly supports this pathway by promoting plasma cell survival via Blimp‐1 activation [61]. In periodontitis, ER stress from inflammatory insults disrupts protein folding, activating the UPR and exacerbating tissue damage [62]. The B Cell Receptor (BCR) Signalling and Cytokine‐Cytokine Receptor Interaction pathways represent key immune activation mechanisms. BCR signalling is enriched for XBP1, HCLS1 and IRF4. HCLS1 acts as a critical scaffold protein [63], IRF4 bidirectionally controls B cell fate [64] and XBP1 is essential for efficient BCR signal transduction [65]. BCR activation drives plasma cell differentiation and pro‐inflammatory cascades (e.g., PI3K‐Akt, NF‐κB), worsening periodontal destruction [66]. The cytokine‐cytokine receptor interaction pathway is enriched for XBP1 and IRF4, with IRF4 acting as a top upstream regulator [67]. XBP1 regulates cytokine secretion and immune cell crosstalk [68]. This pathway, activated by pro‐inflammatory ligands such as IL‐1, recruits immune cells and perpetuates inflammatory responses. Finally, the N‐Glycan Biosynthesis pathway, enriched for XBP1 and IRF4, represents a post‐translational modification process with potential implications for disease progression. XBP1 transcriptionally regulates glycogene expression, altering glycan structures [69], while IRF4 promotes plasma cell differentiation, providing the cellular machinery for N‐glycosylation [70]. Altered N‐glycan patterns may modulate IL‐6 signalling and influence osteoclast activation [71], potentially contributing to bone resorption in periodontitis.

### 4.4 Immune Cells and Key Cells in Periodontitis

To further elucidate how these key genes function within the complex cellular microenvironment of periodontitis, we performed single‐cell analysis, which revealed distinct immune profiles between diseased and healthy periodontal tissues. Diseased tissues showed marked plasma cell infiltration, whereas healthy tissues predominantly contained M2 macrophages, resting dendritic cells and resting mast cells. In healthy periodontal tissues, these cell populations contribute to immune homeostasis through complementary mechanisms: resting dendritic cells maintain immune tolerance and promote regulatory T cell (Treg) differentiation, thereby indirectly restraining osteoclast activity [72]; resting mast cells support mucosal integrity and regulate vascular permeability [73]; and M2 macrophages resolve inflammation, clear cellular debris, inhibit osteoclastogenesis and promote tissue repair. In contrast, diseased tissues exhibited a fundamentally different cellular landscape dominated by plasma cells, whose accumulation reflects chronic immune activation and correlates with ER autophagy‐related gene expression [74]. XBP1 supports plasma cell secretory function through ER expansion [75], while IRF4 serves as a core marker for these cells [76]. Among the diverse cell types identified, T cells and endothelial cells emerged as particularly critical to periodontitis pathogenesis. T cells displayed marked functional heterogeneity: regulatory T cells (Tregs), CD8+ T cells and γδ T cells maintain immune homeostasis, whereas Th1 and Th17 cells drive inflammatory responses and tissue destruction [38]. Endothelial cells exacerbate disease through multiple mechanisms, including interacting with macrophages to release pro‐inflammatory cytokines, presenting antigens to T cells and recruiting immune cells via adhesion molecules [77]. Notably, FOXN1 exhibited high expression in both T cells and endothelial cells, consistent with its established roles in regulating T cell differentiation [78] and potentially influencing T cell trafficking to inflammatory sites [79], thereby impacting both immune cell function and endothelial responses in periodontitis.

Furthermore, pseudotime trajectory analysis of T cells revealed that CD4^+^ T cells were mainly located at the early stage of the differentiation trajectory, while CD8^+^ T cells were enriched at the terminal stage. This distribution pattern may reflect the dynamic process of continuous differentiation of T cells from a naive state to an effector state in the periodontal inflammatory microenvironment [80, 81].

Notably, the expression of HCLS1 exhibited a trend of initial decrease followed by subsequent increase during T cell differentiation, whereas RORA expression declined progressively along the differentiation process. The upregulation of HCLS1 at the late differentiation stage may be associated with its role in maintaining the activation and pro‐inflammatory functions of effector T cells [63]. The persistent downregulation of RORA may weaken its regulatory capacity on T cell differentiation, thereby facilitating Th17 polarization [55].

The dynamic expression changes of these differentiation‐related genes indicate that key genes are involved throughout the transformation of T cells from the naive state to the pro‐inflammatory phenotype and exert distinct regulatory effects at different stages of disease progression.

In addition, endothelial cells presented intense signal crosstalk in the cell–cell communication network. Particularly, the signal exchange between endothelial cells and T cells was markedly enhanced in the disease group, suggesting that endothelial cells may influence the development of periodontitis via interacting with T cells.

Collectively, single‐cell sequencing analysis and immune infiltration analysis jointly demonstrate a disordered protective immune homeostasis and amplified pathogenic immune responses in periodontitis. These findings provide cellular‐level evidence to support the aforementioned neuroendocrine‐immune‐bone metabolism hypothesis.

### 4.5 Therapeutic Potential

To explore therapeutic potential, drug‐gene interaction analysis was done. The result identified XBP1 and RORA as promising targets for pharmacological intervention. Molecular docking studies indicate that MELATONIN, as an XBP1 modulator, exhibits favourable binding properties, and its known anti‐inflammatory and antioxidant characteristics align well with the therapeutic requirements for periodontitis [82]. Mechanistically, MELATONIN can inhibit the NF‐κB signalling pathway, thereby reducing the production of pro‐inflammatory cytokines such as TNF‐α, IL‐1β and IL‐6 [83]. Additionally, it can suppress NLRP3 inflammasome activation—a key mechanism in periodontal tissue destruction. Studies in experimental periodontitis models have confirmed that MELATONIN reduces alveolar bone resorption and gingival inflammation [84].

Nevertheless, it should be emphasized that all the above conclusions are indirect inferences drawn from published literature and computational predictions. Since no functional validation was performed for these drug‐target interactions in the present study, their actual biological relevance cannot be evaluated so far.

No relevant studies on OSM‐S‐85 have been reported in existing literature, and its interaction with RORA also remains at the predictive stage. If future research further verifies the therapeutic potential of these targets, their clinical translation will still be confronted with challenges such as the poor solubility and low oral bioavailability of melatonin [85]. In this context, nanotechnology‐based delivery systems or topical gels could be developed to optimize its pharmacokinetic properties.

Furthermore, it is necessary to explore the potential synergistic effects between melatonin and conventional periodontal therapies. Additionally, the druggability of other key genes including FOXN1 and HCLS1 as well as IRF4 should be systematically assessed. These efforts are expected to provide more abundant candidate directions for the comprehensive development of therapeutic targets for periodontitis.

In conclusion, this study represents a significant advance in understanding periodontitis pathogenesis by establishing a novel neuroendocrine‐immune axis and identifying five pivotal genes, particularly FOXN1 as a previously unrecognized contributor, through integrative bioinformatics and single‐cell transcriptomic analysis. However, it should be noted that the overall findings of this study are highly dependent on data quality, algorithm accuracy and the underlying analytical assumptions. Owing to the complexity and heterogeneity of biological systems, the results may not fully capture the entirety of actual biological processes. Furthermore, the current multi‐level analyses remain relatively preliminary. This study has several limitations. First, although single‐cell analysis clearly indicated that T cells and endothelial cells are the major cellular sources responsible for the differential expression of these key genes, RT‐qPCR performed on bulk tissue extracts fails to provide information regarding cellular localization and spatial distribution at the protein level, making it difficult to further validate the inferences at the cellular level. Second, molecular docking analysis of drug‐gene interactions is merely a computational prediction. Its biological relevance remains to be verified by in vitro and in vivo functional experiments.

Future research will be carried out from the following perspectives. Firstly, immunohistochemistry and immunofluorescence assays will be applied to verify the localization of key genes (especially FOXN1 and RORA) at the protein level, so as to clarify their expression and tissue distribution in target cells such as T cells and endothelial cells. Secondly, a larger clinical cohort will be recruited to validate the predictive performance of the diagnostic nomogram. Thirdly, periodontitis animal models, together with gene knockout and overexpression experiments, will be utilized to systematically elucidate the molecular mechanisms by which these key genes regulate the pathogenesis of periodontitis.

## 5 Conclusion

This study identified five pivotal genes—FOXN1, XBP1, HCLS1, IRF4 and RORA—as key mediators in periodontitis through integrative analysis of transcriptomic data and neuroendocrine‐related genes. These genes collectively participate in critical pathways involving ribosome biogenesis, ER stress response, immune signalling and glycosylation processes. Single‐cell analysis demonstrated that T cells and endothelial cells serve as primary cellular platforms for their pathogenic functions. Drug‐gene interaction analysis identified XBP1 as a druggable target with melatonin showing therapeutic potential. These findings elucidate the neuroendocrine‐immune axis in periodontitis and provide a molecular foundation for developing precision diagnostic and therapeutic strategies.

## Author Contributions

**Jiaguan Zhang:** conceptualization, methodology, data curation, validation, writing – original draft, writing – review and editing, visualization, investigation, funding acquisition, software, resources, formal analysis. **Peishan Huang:** conceptualization, supervision, writing – review and editing. **Ziyan Liu:** data curation, validation, visualization, writing – review and editing. **Huijuan Liu:** project administration, supervision, writing – review and editing. **Jianqing Deng:** visualization, writing – review and editing.

## Funding

This work was supported by Shenzhen Clinical Research Center for Oral Diseases, 20210617170745001.

## Disclosure

Declaration of generative AI use: The authors have nothing to report.

## Ethics Statement

The study was conducted in accordance with the Declaration of Helsinki. The research study has been approved by the IRB of the Eighth Affiliated Hospital, Sun Yat‐sen University. The approval number and date of approval are as follows: [2024‐199‐02] and [2024‐8‐26].

## Consent

The authors have nothing to report.

## Conflicts of Interest

The authors declare no conflicts of interest.

## Supporting information


**Figure S1:** External validation results of machine learning in the GSE10334 dataset. (A) LASSO analysis validation. (B) SVM‐RFE model validation. (C) Venn diagram showing the intersection of feature genes selected by LASSO and SVM‐RFE in the validation set.

**Table S1:** 1441 retrieved neuroendocrine‐related genes (NRGs) from a published study.

**Table S2:** Primers for key genes.

**Table S3:** Significant Enrichment of 9 Candidate Genes in 91 GO Terms: 75 Biological Processes (BPs) and 16 Molecular Functions (MFs).

**Table S4:** Enrichment of FOXN1 in 139 Pathways: Top 5 Pathways (Ribosome, Haematopoietic Cell Lineage, Cytokine‐Cytokine Receptor Interaction, Viral Protein Interaction with Cytokine and Cytokine Receptor, Neuroactive Ligand‐Receptor Interaction).

## Data Availability

The datasets analysed in this study are available in the [Gene Expression Omnibus (GEO) database] repository [https://www.ncbi.nlm.nih.gov/gds], including GSE16134, GSE223924 and GSE171213 datasets.
